# Translation and evaluation of the HeartQoL in patients with coronary heart disease in Iceland

**DOI:** 10.1186/s12955-023-02161-7

**Published:** 2023-08-09

**Authors:** Margrét Hrönn Svavarsdóttir, Brynja Ingadottir, Neil Oldridge, Kristofer Årestedt

**Affiliations:** 1 Faculty of Nursing, University of Akureyri, Nordurslod 2, Solborg, Akureyri, 600 Iceland; 2 Faculty of Nursing and Midwifery, University of Iceland, Reykjavik, Iceland; 3 Landspitali University Hospital, Reykjavik, Iceland; 4 College of Health Sciences, University of Wisconsin-Milwaukee, Milwaukee, WI USA; 5 Faculty of Health and Life Sciences, Linnaeus University, Kalmar, Sweden; 6 Department of Research, Region Kalmar County, Kalmar, Sweden

**Keywords:** Heart disease, Patient-reported outcome measures, Health-related quality of life, Validation, Reliability, Validity

## Abstract

**Background:**

Health-related quality of life (HRQoL) reflects an individual’s own perception of their symptom burden, functional limitations, prognosis, overall health and changes associated with treatment. The HeartQoL is a validated heart disease-specific questionnaire with a physical and an emotional subscale that is used internationally to assess HRQoL in patients with coronary heart disease (CHD). The aim of this study was to translate and evaluate the psychometric properties of the HeartQoL in patients with CHD in Iceland.

**Methods:**

Patients ≥ 18 years (n = 396; mean age 64.4 ± 8.8 years; 79.6% male) admitted with CHD were recruited from two hospitals in Iceland and completed the Icelandic versions of the HeartQoL, Short-Form 12v2 Health Survey (SF-12v2), and Hospital Anxiety and Depression Scale (HADS). A subsample of 47 patients completed the HeartQoL 14 days later. Confirmatory factor analysis for ordinal data was used to evaluate the measurement model with a physical and an emotional subscale. Convergent and divergent validity, internal consistency, and test-retest reliability were evaluated.

**Results:**

Overall, the hypothesized two-factor structure of the Icelandic version of the HeartQoL was supported. However, problems with cross-loadings and correlated error variances were identified. Convergent and divergent validity were supported in correlational analyses between HeartQoL, SF-12v2, and HADS. Internal consistency reliability, measured by ordinal alpha, was good for the physical (*α* = 0.96) and emotional (*α* = 0.90) subscale. According to intraclass correlations (ICC), acceptable test-retest reliability was demonstrated (ICC = 0.79–0.86).

**Conclusion:**

With the two-factor structure confirmed, the Icelandic HeartQoL demonstrated satisfactory psychometric properties in the sample of patients with CHD. Users of the instrument can use the original scoring.

**Supplementary Information:**

The online version contains supplementary material available at 10.1186/s12955-023-02161-7.

## Background

Cardiovascular disease remains the most common cause of disease burden in Europe, with coronary heart disease (CHD) its most common manifestation [1]. Patient-centered outcomes research emphasizes the importance of outcomes that patients notice and are aware of such as symptoms, functional status, and health-related quality of life (HRQoL). Thus, patient perceived health status is an important measure of health [2], reflecting the effects of disease and treatment from the patient’s own perspective so helping to identify health-related problems, to facilitate communication with patients, to follow the trajectory of disease and to evaluate intervention and treatment effects [3, 4].

To identify patient needs and evaluate intervention and treatment effects, HRQoL instruments, which come in two major designs — generic and disease-specific — need to be reliable, valid, and responsive [3–6]. As generic instruments address multiple aspects of HRQoL across a broad spectrum of diseases or patient groups, they are less specific and have poorer sensitivity than disease-specific instruments [7].

Coronary heart disease-specific HRQoL instruments have been designed for patients with heart failure, arrhythmias, angina pectoris, and myocardial infarction [8] with more than one diagnosed heart condition complicating the decision of which disease-specific HRQoL instrument to use. To overcome this problem, core disease-specific instruments have been developed to cover different heart disease diagnoses [8]. The HeartQoL was developed as a core CHD disease-specific HRQoL instrument in patients with angina pectoris, myocardial infarction, and ischemic heart failure. The instrument includes 14 items with a 10-item physical and a 4-item emotional subscale [9, 10].

The HeartQoL has been psychometrically evaluated in 11 peer-reviewed publications in patients speaking at least one of 27 European languages, Mandarin Chinese, Persian (Farsi) and Malay (Bahasa Malaysia) [11]. The dimensionality of the HeartQoL has mainly been evaluated using Mokken scale analysis or factor analysis. While the Mokken scale analyses have confirmed the suggested two factor model [1–6], evaluations with exploratory and/or confirmatory factor analyses have shown some problems with cross-loadings, correlated error variances, and/or poor model fit in different language versions [7–9, 12–14]. One problem with Mokken scale analysis is that only the Loevinger’s H coefficient has been used to justify scalability. Thus, potential issues with correlated error variances or local dependency are unknown based on these studies. Further, as the Mokken scale analysis is conducted on each subscale individually, potential issues with cross-loadings are not addressed. From that perspective, confirmatory factor analysis (CFA) models taking the ordinal nature of data into account, or multidimensional item response theory models, seem to be the most appropriate methods to use in future evaluations of the HeartQoL.

Translations of HRQoL instruments is another critical aspect that can have serious validity and reliability consequences. A poor translation process leading to a questionnaire that is not equivalent to the original language questionnaire limits the comparability of responses across populations with different languages or cultures [15]. Therefore, aim of this study was to translate and evaluate the psychometric properties of the HeartQoL in patients with CHD in Iceland.

## Methods

### Participants

Patients with CHD were recruited from two main hospitals in Iceland between October 2017 and May 2019 for this psychometric study. All patients ≥ 18 years who were admitted electively or acutely for CHD (angina, acute myocardial infarction, acute myocardial ischemia, elective percutaneous coronary intervention and/or coronary artery bypass graft) were eligible for the study. Information on age, sex, diagnosis, and treatment was collected from medical records. Eligible patients were asked to complete a battery of questionnaires at discharge. Patients who did not speak or write Icelandic and those unable or unwilling to respond to the questionnaire were excluded from the study.

A total of 446 patients consented to participate in the study; 50 patients had complete missing HeartQoL data, and the final sample included 396 patients (88% response rate). The mean age was 64.4 (*SD* = 8.8) years; 315 (79.6%) were males; most patients were cohabiting (n = 291, 73.5%) and had at least upper secondary education (n = 259, 68.3%). A subsample (n = 47), of these 396 patients also completed the HeartQoL after 14 days to evaluate test-retest reliability. Patients who completed the test-retest assessment were significantly more often men (*p* = 0.031) with no age, cohabitation, education, or admission diagnosis differences. Detailed information about the sample is presented in Table 1.

**Table 1:** Sample characteristics (*n* = 396)

		Test-retest		
	All	No	Yes	*p*-value
**Age** (years), *M* (*SD*)	64.4 (8.8)	64.3 (8.9)	64.6 (7.9)	0.867 ^a^
**Sex**, *n* (%)				0.031 ^b^
Women	81 (20.5)	77 (22.1)	4 (8.5)	
Men	315 (79.6)	272 (77.9)	43 (91.5)	
**Cohabitation**, *n* (%)				0.116 ^b^
Cohabit	291 (73.5)	252 (72.2)	39 (83.0)	
Living alone	105 (26.5)	97 (27.8)	8 (17.0)	
**Education**, *n* (%)				0.362 ^b^
Elementary school	120 (31.7)	109 (32.7)	11 (23.9)	
Upper secondary school	171 (45.1)	146 (43.8)	25 (54.4)	
University	88 (23.2)	78 (23.4)	10 (21.7)	
Missing data	17	16	1	
**Admission diagnosis**, *n* (%)				0.329 ^b^
Acute coronary syndrome	45 (11.4)	41 (11.8)	4 (8.5)	
ST-elevation myocardial infarction	87 (22.0)	72 (20.6)	15 (31.9)	
Non-ST-elevation myocardial infarction	61 (15.4)	52 (14.9)	9 (19.2)	
Elective percutaneous coronary intervention	167 (42.2)	152 (43.6)	15 (31.9)	
Elective coronary artery bypass graft	36 (9.1)	32 (9.2)	4 (8.5)	

^a^Unpaired t-test

^b^Chi-square test

### Instruments

Consenting patients completed demographic questions, the HeartQoL to assess disease-specific HRQoL, the Short-Form 12v2 Health Survey (SF-12v2) [16] to assess generic HRQoL, and the Hospital Anxiety and Depression Scale (HADS) [17, 18] to assess symptoms of anxiety and depression.

#### HeartQoL

The 14-item HeartQoL is divided into two subscales, a 10-item physical and a 4-item emotional subscale. All 14 items are scored on a 4-point Likert-type scale ranging between 0 (‘A lot’) and 3 (‘No’). The subscale scores are calculated as the mean rating across the items with higher scores indicating better HRQoL [9]. The HeartQoL was translated and culturally adapted from English to Icelandic using standard procedures as described by Ware (1995) [19] and the Medical Outcomes Trust committee (2002) [5]. Two individuals, one a health care professional, the other not, both fully bilingual in Icelandic (as their first language) and English independently translated the HeartQoL into Icelandic. The backtranslation into English was conducted by two different translators, one a healthcare professional, the other not, both also fully bilingual in Icelandic and English and blinded to the original version. The back-translations were sent to the developer of the HeartQoL who compared them to the original English version of the HeartQoL. Items not accurately translated were discussed and re-translated by the research group in collaboration with the developer of the instrument until a consensus was reached. The Icelandic version of the HeartQoL can be found in Appendix.

#### Short-Form 12v2 Health Survey

The SF 12v2 is a 12-item, generic HRQoL instrument designed to measure health status from the patient’s perspective and covers eight health dimensions: Physical functioning (PF), Role physical (RP), Bodily pain (BP), General health (GH), Vitality (VT), Social functioning (SF), Role emotional (RE), and Emotional health (EH). Based on a scoring algorithm, each dimension has a possible score range between 0 and 100 with higher scores indicating better HRQoL. In addition, two component summary scores can be calculated, one each for physical (PCS-12) and mental (MCS-12) health [16]. The instrument has been shown to be reliable and valid for use in patients with CHD [20]. The Icelandic version of the SF-36, from which the SF-12v2 was derived, has acceptable psychometric properties [21].

#### Hospital Anxiety and Depression Scale

The 14-item HADS is designed to screen for anxiety and depression. Each item is scored on a response scale ranging between 0 and 3, with subscale scores between 0 and 21 with higher scores implying more problems [17, 18]. The Icelandic version of the HADS has demonstrated satisfactory psychometric properties among university students [22]. Evaluated with ordinal alpha, both subscales demonstrated good internal consistency in the present study, 0.90 for anxiety and 0.88 for depression.

### Data analysis

Descriptive statistics were used to present the sample, mean and standard deviations for continuous normally distributed data, median and quartiles for ordinal data, and frequencies for nominal data. Unpaired t-test and chi-square test were used to compare patients who did and did not complete the test-retest assessment. To consider the ordinal nature of item and scale scores, all analyses, except for the evaluation of test-retest reliability, were based on non-parametric statistics.

Item analysis was used to evaluate the basic measurement properties on an item level [23]. The score distribution was evaluated using median, quartiles, frequencies, skewedness, and kurtosis statistics. A normal distribution has a skewness and kurtosis value close to 0. Item discrimination was evaluated using item-total correlations adjusted for overlap based on polychoric (polyserial) correlations (*r*_*pc*_). Higher item-total correlations imply a higher discriminating capacity and should therefore be at least > 0.2 [24] with higher cut-off levels also reported in the literature.

The factor structure was evaluated using CFA for ordered category indicator variables, that is, the weighted least squares means and variance adjusted (WLSMV) method, and polychoric correlations [25]. Two CFA models were considered: a baseline two-factor model, and a modified two-factor model. The model fit was evaluated in terms of factor loadings and goodness-of-fit indices including the Root Mean Square Error of Approximation (RMSEA) with 90% confidence interval (*CI*), Standardised Root Mean Square Residual (SRMR), Comparative Fit Index (CFI) and Tucker-Lewis Index (TLI). Satisfactory model fit was defined according to Hu and Bentler [26] with RMSEA close to 0.06 or below, CFI and TLI close to 0.95 or greater and SRMR close to 0.08 or below. Missing data were treated with listwise deletion since the WLSMV estimation cannot handle these, in contrast to the full information maximum likelihood (FIML).

Convergent and divergent validity were examined simultaneously using the multitrait-multimethod (MTMM) approach [27]. Spearman correlations (*r*_*s*_) were used to estimate associations between the HeartQoL physical and emotional subscales and the PCS-12 and MCS-12 subscales. To support convergent and divergent validity, the strongest correlations are expected between similar constructs measured by different methods (HeartQoL physical subscale and PCS-12 and HeartQoL emotional subscale and MCS-12), while the weakest correlations are expected between different constructs measured by different methods (HeartQoL physical subscale and MCS-12 and HeartQoL emotional subscale and PCS-12). Convergent and divergent validity were also evaluated by correlating the HeartQoL scales with the eight health dimensions of the SF-12v2 and HADS subscales of anxiety and depression. To support convergent and divergent validity, the HeartQoL physical subscale should correlate more strongly with the SF-12v2 physical dimensions (PF, RP, BP and GH) while the HeartQoL emotional scale should correlate more strongly with the SF-12v2 mental dimensions (VT, SF, RE and ME). Further, the HeartQoL emotional subscale should correlate more strongly with symptoms of anxiety and depression measured by the HADS compared to the HeartQoL physical subscale. All analyses of the convergent and divergent validity were based on the original measurement model of the HeartQoL.

An ordinal version of Cronbach’s alpha for Likert-type scales was used to estimate the internal consistency reliability on the original measurement model of the HeartQoL [28]. Ordinal alpha has the same interpretation as the traditional Cronbach’s alpha coefficient, i.e., > 0.7 was considered satisfactory [24, 28]. Cronbach’s alpha was also calculated to facilitate comparisons with previous studies. Component reliability was estimated with ordinal omega and calculated for both the bassline CFA model and the modified CFA model [29].

Test-retest reliability of the scale scores, based on the original measurement model of the HeartQoL, was evaluated using intraclass correlations (*ICC*) (two-way mixed effect, single measurement, absolute agreement); values between 0.50 and 0.75, 0.75 and 0.90 and > 0.90 indicate moderate, good and excellent reliabilities, respectively [30].

Statistical significance was set at *p* < 0.05. All analyses were conducted with R 4.2.3 (R Foundation for Statistical Computing, Vienna, Austria), including the following packages: irr 0.84.1, lavaan 0.6–13, psych 2.2.9, semTools 0.5–6, sjmisc 2.8.9, and summarytools 1.0.1.

### Ethical considerations

The study was approved by the National Bioethics Committee for Medical Research Ethics (17–159) and permission to examine patient records was granted by hospital authorities. The study was conducted in accordance with the Helsinki Declaration [31].

## Results

### Item analysis and score distribution

The HeartQoL items demonstrated either a discrete uniform distribution (items 3–6, 8 and 13) or a positive skewed distribution with ceiling effects (1, 2, 7, 9–12 and 14). The observed inter-item correlations (*r*_*pc*_) were between 0.74 and 0.88 for the physical subscale and 0.74–0.81 for the emotional subscale, indicating good discrimination. Missing data among the items was low (2–4%) with item 13 not answered by 11% of the patients (Table 2). Both subscales demonstrated a negative skewed distribution, somewhat more pronounced for the emotional scale than the physical scale (skewness = -0.67 vs. -0.37) (Table 3).

**Table 2:** HeartQoL item analysis (*n* = 396)

		Item score distribution, %								
“Have you been bothered by …”	*Median* (*Q1, Q3*)	No 3	Little 2	Some 1	A lot 0	Missing	Skew ^a^	Kurt ^a^	ITC ^b^	α item deleted ^c^
**Physical subscale**										
1. Walking indoors on level ground?	3 (2, 3)	72	12	11	2	2	-1.69	1.66	0.74	0.95
2. Gardening, vacuuming, or carrying groceries?	3 (1, 3)	51	17	18	11	3	-0.77	-0.84	0.86	0.95
3. Climbing a hill or a flight of stairs without stopping?	2 (1, 3)	30	21	28	20	2	-0.08	-1.38	0.86	0.95
4. Walking more than 100 yards at a brisk pace?	2 (1, 3)	34	18	26	17	4	-0.19	-1.39	0.85	0.95
5. Lifting or moving heavy objects?	2 (1, 3)	35	21	25	16	3	-0.28	-1.30	0.81	0.95
6. Feeling short of breath?	1 (1, 2)	19	26	38	15	2	0.13	-1.01	0.77	0.95
7. Being physically restricted?	2 (1, 3)	37	25	28	7	4	-0.36	-1.09	0.83	0.95
8. Feeling tired, fatigued, low on energy?	1 (1, 2)	15	24	38	20	4	0.25	-0.93	0.76	0.95
13. Being limited in doing sports or exercise?	2 (1, 3)	27	18	26	19	11	-0.04	-1.40	0.68	0.95
14. Working around the house or yard?	2 (1, 3)	43	22	22	9	4	-0.58	-0.99	0.88	0.95
**Emotional subscale**										
9. Not feeling relaxed and free of tension?	2 (1, 3)	39	25	25	6	4	-0.46	-1.02	0.74	0.88
10. Feeling depressed?	3 (2, 3)	61	21	13	2	3	-1.27	0.54	0.81	0.85
11. Being frustrated?	2 (2, 3)	42	30	20	4	4	-0.62	-0.70	0.77	0.87
12. Being worried?	2 (1, 3)	35	32	23	7	2	-0.47	-0.83	0.77	0.87

^a^ A normal distribution has a skewness and kurtosis value close to 0

^b^ Item-total correlations based on polyserial correlations, corrected for overlap

^c^ Ordinal alpha if item deleted

**Table 3:** Score distribution for the HeartQoL, SF-12v2, and HADS (*n* = 325–390)

Scales	*Median* (*Q1, Q3*)	Skew ^a^	Kurt ^a^
**HeartQoL**			
Physical subscale	1.9 (1.3, 2.6)	-0.37	-0.90
Emotional subscale	2.3 (1.8, 2.8)	-0.67	-0.38
**SF-12v2**			
Physical functioning	50 (25, 75)	0.08	-1.16
Role physical	50 (25, 75)	0.19	-0.97
Bodily pain	75 (50, 100)	-0.59	-0.69
General health	60 (25, 60)	0.08	-1.15
Vitality	50 (25, 75)	0.15	-0.84
Social function	75 (50, 100)	-0.64	-0.83
Role emotional	63 (50, 100)	-0.36	-0.80
Mental health	75 (56, 88)	-0.69	-0.09
Physical Component Summary (PCS-12)	41.2 (34.1, 47.8)	-0.14	-0.47
Mental Component Summary (MCS-12)	50.3 (42.2, 57.3)	-0.39	-0.42
**HADS**			
Anxiety	4 (2, 7)	0.80	0.10
Depression	3 (1, 6)	1.07	0.91

^a^A normal distribution has a skewness and kurtosis value close to 0

### Factor structure

Factor loading for the two-factor baseline model ranged between 0.74 and 0.91 for the physical and 0.74–0.93 for the emotional subscale (Table 3). While the CFI, TLI, and SRMR indicated satisfactory model fit, the RMSEA did not (Table 4). Inspections of the standardized residual covariance matrix (the difference between the model implied covariance matrix and the sample covariance matrix) and the modification index identified problems with cross-loadings for items 8 and 9 and correlated error variances between items 3 and 4. The suggested revisions are conceptually defendable; items 8 and 9 reflect both physical and emotional health while item 3 and 4 capture physical efforts but at different levels. Consequently, a modified two-factor model was examined. All fit indices improved with the RMSEA close to the suggested criteria of 0.06. Additionally, the RMSEA did not deviate significantly from the criteria of 0.05 (*p* = 0.085) and the 90% *CI* covered both the 0.05 and 0.06 criteria (Tables 4 and 5).

**Table 4:** Standardized factor loadings, error variances (brackets), factor correlations, and reliability estimates for the HeartQoL (*n* = 314)

		Two-factor baseline model			Two-factor modified model^a^	
Items		Physical subscale	Emotional subscale		Physical subscale	Emotional subscale
**Physical subscale**						
1. Walk indoors on level ground?		0.79 (0.38)			0.80 (0.37)	
2. Garden, vacuum, or carry groceries?		0.87 (0.24)			0.88 (0.23)	
3. Climb a hill or a flight of stairs without stopping?		0.89 (0.20)			0.86 (0.26)	
4. Walk more than 100 yards at a brisk pace?		0.88 (0.22)			0.85 (0.28)	
5. Lift or move heavy objects?		0.85 (0.28)			0.86 (0.26)	
6. Feeling short of breath?		0.78 (0.40)			0.79 (0.38)	
7. Being physically restricted?		0.85 (0.29)			0.85 (0.28)	
8. Feeling tired, fatigued, low on energy?		0.81 (0.34)			0.70 (0.34)	0.21 (0.34)
13. Being limited in doing sports or exercise?		0.74 (0.45)			0.75 (0.44)	
14. Working around the house or yard?		0.91 (0.18)			0.91 (0.17)	
**Emotional subscale**						
9. Not feeling relaxed and free of tension?			0.93 (0.14)		0.24 (0.30)	0.70 (0.30)
10. Feeling depressed?			0.86 (0.27)			0.91 (0.18)
11. Being frustrated?			0.74 (0.45)			0.77 (0.40)
12. Being worried?			0.78 (0.39)			0.83 (0.31)
**Factor correlation**		0.57			0.47	
**Reliability**						
Ordinal alpha		0.96	0.90			
Traditional Cronbach’s alpha		0.93	0.84			
Composite reliability		0.91	0.85		0.85	0.63

Cross-loadings for items 8 and 9, correlated residual variances between items 3 and 4 (0.49)

^a^Items 8 and 9 as indicator variables of both physical and emotional health and correlated residual variances between items 3 and 4

**Table 5:** Goodness-of-fit indices for the confirmatory factor analyses of the HeartQoL *(n* = 314)

	*χ*^*2*^ goodness-of-fit			RMSEA						
Model	*χ*^*2*^ (*df*)	*p*-value		RMSEA	90% *CI*	*p*-value		CFI	TLI	SRMR
**Two-factor baseline model**	261.7 (76)	< 0.001		0.088	0.077, 0.100	< 0.001		0.977	0.972	0.060
**Two-factor modified model** ^**a**^	157.4 (73)	< 0.001		0.061	0.048, 0.074	0.085		0.990	0.987	0.043

^a^Items 8 and 9 as indicator variables of both physical and emotional health and correlated residual variances between items 3 and 4

### Convergent and divergent validity

The MTMM analysis supported convergent and divergent validity. As expected, the strongest correlations were found between the similar constructs measured with different scales (*r*_*s*_ = 0.73 and 0.63) with the weakest correlation between dissimilar constructs measured with different scales (*r*_*s*_ = 0.35 and 0.29) (Table 6).

**Table 6:** Multitrait-multimethod (MTMM) based on Spearman rank order correlation coefficients between the HeartQoL physical and emotional subscales and the SF-12v2 physical and mental component scores (*n* = 286)

	Physical scales			Emotional/Mental scales	
	HeartQoL	SF-12v2		HeartQoL	SF-12v2
**Physical scales**					
HeartQoL					
SF-12v2	0.73^*** a^				
**Emotional/Mental scales**					
HeartQoL	0.46^*** b^	0.29^*** c^			
SF-12v2	0.35^*** c^	0.17^** b^		0.63^*** a^	

**p < 0.01, ***p < 0.001

a = Same construct measured by different scales; b = Different constructs measured by same scales; c = Different constructs measured by different scales

The correlations between the HeartQoL, SF-12v2, and HADS supported both convergent and divergent validity. As expected, the HeartQoL physical subscale correlated more strongly with the SF-12v2 physical health dimensions (i.e., PF, RP, and BP; *r*_*s*_ = 0.58–0.67) than with the mental dimensions (i.e., SF, RE, and EH; *r*_*s*_ = 0.29–0.47). Similarly, the HeartQoL emotional subscale correlated more strongly with the SF-12v2 mental health dimensions (*r*_*s*_ = 0.49–0.65) than with the physical dimensions (*r*_*s*_ = 0.28–0.39). Furthermore, the HeartQoL emotional subscale correlated more strongly with HADS symptoms of anxiety and depression than the HeartQoL physical subscale (*r*_*s*_ = -0.69 and − 0.59 and *r*_*s*_ = -0.34 and − 0.55, respectively). Despite the expected correlation pattern, the differences between the two subscales were minor regarding the SF (*r*_*s*_ = 0.47 vs. 0.50) and RE (*r*_*s*_ = 0.42 vs. 0.49) in the SF-12v2 and HADS depression (*r*_*s*_ = -0.55 vs. -0.59) (Table 7).

**Table 7:** Convergent and divergent validity based on Spearman rank order correlation coefficients between the HeartQoL subscales and SF-12v2 health dimensions and HADS subscales (*n* = 286)

	SF-12v2 health dimensions									HADS subscales	
	PF	RP	BP	GH	VT	SF	RE	EH		Anxiety	Depression
**HeartQoL physical subscale**	0.60	0.67	0.58	0.55	0.63	0.47	0.42	0.29		-0.34	-0.55
**HeartQoL emotional subscale**	0.28	0.39	0.39	0.41	0.42	0.50	0.49	0.65		-0.69	-0.59

All correlations are significant to a level at *p* < 0.001

PF = Physical functioning, RP = Role physical, BP = Bodily pain, GH = General health, VT = Vitality, SF = Social functioning, RE = Role emotional, EH = Emotional health, HADS = Hospital Anxiety and Depression Scale

### Reliability

Internal consistency reliability, as measured by ordinal alpha coefficient, was high for the HeartQoL physical (0.96) and emotional (0.90) subscales. The corresponding traditional Cronbach’s alpha values were 0.93 and 0.84, respectively. Composite reliability was 0.96 and 0.90 for the physical and emotional subscales in the baseline CFA model and 0.85 and 0.63 in the modified CFA model (Table 4).

Test-retest reliability was good for the HeartQoL physical (*ICC* = 0.84, 95% *CI* = 0.72, 0.91, *n* = 46) and emotional (*ICC* = 0.78, 95% *CI* = 0.64, 0.88, *n* = 47) subscales.

## Discussion

Following translation the present study evaluated the psychometric properties of the Icelandic version of the HeartQoL taking the ordinal nature of data into account. The two-factor structure was confirmed overall although, by using the CFA models, some problems with cross-loading and correlated error variances were identified. In addition, the instrument demonstrated good construct validity and reliability in the present sample of patients with CHD.

Overall, missing data were small except for HeartQoL item 13 with 11% missing values suggesting that the meaning of exercise in the Icelandic language may focus on elite sport or intense training and this should be considered in future revisions and evaluations of the Icelandic version of the HeartQoL. In case of missing data for this item, users of the Icelandic version of the HeartQoL may calculate the mean score without this item.

The two-factor model generally demonstrated an acceptable model fit although cross-loading for items 8 and 9 and correlated errors between items 3 and 4 were observed. The problem with cross-loadings for items 8 and 9 has been reported in previous HeartQoL studies [14, 32] and may be because they are not as clear indicators for physical and emotional attributes as the other indicators in the respective subscale. Problems with correlated errors have not been widely reported although they have been reported in the Persian HeartQoL [10, 13] which, among other reasons, can arise from overlapping items measuring the same attribute and multidimensionality [25]. The problem with correlated errors between items 3 and 4 may be because both items measure physical efforts but at different levels. The hypothesized dimensionality of the HeartQoL has been evaluated using Mokken scale analysis in patients with CHD; for example, the original HeartQoL validation study [9] and the evaluations of both the German [33] and Italian [34] version of the HeartQoL concluded that the global scale and the two subscales are unidimensional based solely on the Loevinger H coefficient. Thus, the assumptions of unidimensionality need to be further explored in future evaluation studies of the HeartQoL.

Convergent and divergent validity of the HeartQoL were supported in the correlation analyses including the MTMM analysis and the correlations with the eight health dimensions of the SF-12v2 and the HADS subscales. Although the HeartQoL subscales correlated significantly with all other scales in the present study, no correlation was above 0.70. This is not an unexpected finding, since HeartQoL is a disease-specific instrument while SF-12v2 and HADS are generic instruments with the measures expected to overlap, but not strongly. However, these findings underpin the importance of using both generic and disease-specific instruments to measure HRQoL in patients with CHD. That the physical and emotional subscale in the HeartQoL correlated equally strongly with HADS depression as well as with the SF and RE in SF-12v2 was unexpected. This finding is somewhat hard to explain as the differences, as expected, were more pronounced for the EH in SF-12v2 and HADS anxiety.

Both HeartQoL subscales demonstrated high internal consistency reliability measured by both ordinal alpha and Cronbach’s alpha. It should be noted that the ordinal alpha was higher than Cronbach’s alpha illustrating the importance of taking the ordinal nature of the data into account [35]. The internal consistency measured by Cronbach’s alpha was consistent with levels that have been reported in a study of 22 European languages [14], in English [10], German [33] and Italian [34]. Despite satisfactory high alpha values on the raw scores, these reliability coefficients should be interpreted with caution in models with a bad fit [25]. Since ordinal and Cronbach’s alpha are based on raw scores, that is, the models without cross-loadings and correlated error variances, these results have probably overestimated the reliability of the HeartQoL scales. On the contrary, the composite reliability corrects the alpha’s overestimation bias in multidimensional data and correlations between error variances [36] which may explain why the composite reliability coefficients differed from the ordinal and Cronbach’s alpha in the modified two-factor model.

Test-retest reliability was good for the HeartQoL subscales. However, the lower limit of the 95% CI for the emotional subscale was below 0.75, indicating that moderate rather than good test-retest reliability could not be excluded. Compared to a study by Lee et al. [37] we observed a higher test-retest reliability in the physical subscale with similar levels in the emotional subscale. However, Lee et al. [37] did not report the CI; therefore, no strong conclusions can be drawn based on these differences.

The translation of the HeartQoL followed recommended procedures for patient-reported outcome measures [38] using the same standard forward and backward translations and pilot testing that were previously used in translating the HeartQoL from English into other languages [11]. We therefore argue that the Icelandic version linguistically is equivalent to the original English version of the questionnaire. However, as problems with missing data were detected for item 13, further exploration of this item, for example with cognitive interviews, is recommended. In addition, to make valid and meaningful comparisons between different countries and cultures, evaluation of measurement invariance across different language versions are needed. Until more evidence is obtained about the findings from the present study, the Icelandic version of the HeartQoL should be used as recommended.

### Study strengths and limitations

The HeartQoL questionnaire is a heart disease-specific HRQoL instrument for patients with CHD and has been validated internationally in 30 languages in 11 peer-reviewed publications [10, 12–14, 33, 34].

Strengths of this study include the population of patients with angina, acute myocardial infarction, acute myocardial ischemia, elective percutaneous coronary intervention and/or coronary artery bypass graft. The rigorous translation process and the use of appropriate statistical methods, such as CFA models for ordinal data, are also strengths of this study. This is important since parametric methods tend to underestimate the association between indicator variables, increase the risk for identifying pseudo factors, and create incorrect test statistics and standard errors [25].

Limitations of our study include the fact that, as this is a sub-study from a larger empirical study, no priori sample size calculation was conducted. Further, because of the restricted sample size with each diagnosis, we analyzed the group of patients with CHD as a single cohort. However, the sample size of 396 patients was considered large enough with 10–20 observations for each free parameter in the model [39] and prior simulation studies have shown that the WLSMV performs well in small samples [25]. However, these limitations mean that the measurement properties within specific cardiac diagnoses need to be addressed in future validation studies of the Icelandic HeartQoL. Finally, the proportion of women who completed the test-retest assessment was smaller than for those who did not, and the opposite situation was found for the males. On the other hand, no significant differences were found in age, cohabitation, education, and admission diagnosis. We can only speculate why women were underrepresented in the test-retest assessment, but a reasonable explanation may be that they were significantly older compared with the men (*M* = 66.6 vs. 63.8, *t*(394) = 2.62, *p* = 0.009).

## Conclusion

The Icelandic version of the HeartQoL has sound measurement properties in patients with CHD. Users of the Icelandic HeartQoL can use the original scoring but should be aware of problems with cross-loadings and correlated error variances which may increase measurement errors for the physical and emotional subscales.

### Electronic supplementary material

Below is the link to the electronic supplementary material.

Supplementary Material 1

## Data Availability

The datasets used and/or analyzed during the current study are available from the corresponding author on reasonable request.

## Abbreviations

BP: Bodily pain
CFA: Confirmatory factor analysis
CFI: Comparative Fit Index
CHD: Coronary heart disease
EM: Emotional health
GH: General health
HADS: Hospital Anxiety and Depression Scale
HRQoL: Health-related quality of life
MCS: Mental component scores
MTMM: Multitrait-multimethod approach
PCS: Physical component scores
PF: Physical functioning
RE: Role emotional
RMSEA: Root Mean Square Error of Approximation
RS: Role physical
SF-12v2: Short-Form 12v2 Health Survey
SF: Social functioning
SRMR: Standardized Root Mean Square Residual
TLI: Tucker-Lewis Index
VT: Vitality
WLSMV: Weighted least squares means and variance adjusted
