# Supplementary material for: Translation and evaluation of the HeartQoL in patients with coronary heart disease in Iceland
Source: Health Qual Life Outcomes. 2023 Aug 9;21:84. doi: 10.1186/s12955-023-02161-7 (PMC10410800; doi:10.1186/s12955-023-02161-7)
Supplement: Supplementary file 1 — Supplementary Material 1 [file 12955_2023_2161_MOESM1_ESM.pdf]

Við þökkum þér fyrir að svara eftirfarandi spurningum en þær munu veita okkur skilning á hvernig hjartasjúkdómurinn þinn hefur áhrif á þig.

Okkur langar að vita hvernig hjartasjúkdómurinn þinn hefur truflað þig og hvernig þér hefur liðið **SÍÐASTLIÐNAR 4 VIKUR.**

Vinsamlegast dragðu hring um eina tölu

Í fyrsta lagi, síðastliðnar 4 vikur, hefur þú átt í erfiðleikum við:	Nei	Lítið	Nokkuð	Mikið
1. Að ganga innandyra á jafnsléttu?	3	2	1	0
2. Garðvinnu, að ryksuga, eða bera innkaupapoka?	3	2	1	0
3. Að ganga upp brekku eða stiga án þess að stoppa?	3	2	1	0
4. Að ganga rösklega lengra en 100 metra?	3	2	1	0
5. Að lyfta eða færa til þunga hluti?	3	2	1	0

Í öðru lagi, síðastliðnar 4 vikur, hefur þú fundið fyrir:	Nei	Lítið	Nokkuð	Mikið
6. Mæði?	3	2	1	0
7. Takmörkun á hreyfigetu?	3	2	1	0
8. Þreytu, magnleysi, orkuleysi?	3	2	1	0
9. Að geta ekki slakað á eða losnað við spennu?	3	2	1	0
10. Þunglyndi?	3	2	1	0
11. Að vera pirruð/pirraður?	3	2	1	0
12. Að vera áhyggjufull(ur)?	3	2	1	0
13. Takmörkun við íþróttaiðkun eða þjálfun?	3	2	1	0
14. Erfiðleikum við heimilistörf eða við garðvinnu?	3	2	1	0

Kærar þakkir

© Copyright ESC 2012

HeartQoL questionnaire sample, subject to licensing – visit www.escardio.org for permission / licenses.
